# Fuzzy Index to Evaluate Edge Detection in Digital Images

**DOI:** 10.1371/journal.pone.0131161

**Published:** 2015-06-26

**Authors:** Felicitas Perez-Ornelas, Olivia Mendoza, Patricia Melin, Juan R. Castro, Antonio Rodriguez-Diaz, Oscar Castillo

**Affiliations:** 1 School of Engineering, Autonomous University of Baja California, Tijuana, Baja California, Mexico; 2 Division of Graduate Studies, Tijuana Institute of Technology, Tijuana, Baja, California, Mexico; University California Los Angeles, UNITED STATES

## Abstract

In literature, we can find different metrics to evaluate the detected edges in digital images, like Pratt's figure of merit (FOM), Jaccard’s index (JI) and Dice’s coefficient (DC). These metrics compare two images, the first one is the reference edges image, and the second one is the detected edges image. It is important to mention that all existing metrics must binarize images before their evaluation. Binarization step causes information to be lost because an incomplete image is being evaluated. In this paper, we propose a fuzzy index (FI) for edge evaluation that does not use a binarization step. In order to process all detected edges, images are represented in their fuzzy form and all calculations are made with fuzzy sets operators and fuzzy Euclidean distance between both images. Our proposed index is compared to the most used metrics using synthetic images, with good results.

## Introduction

In recent years, a wide variety of image taking devices has been developed. Thus, an advance in image processing techniques has become a major interest for many researchers. Although much has been accomplished, there is still a lot to do on this field.

Image processing methods are complex, and widely used in a multitude of areas such as medicine, military, geographical, just to name a few [1], [2], [3].

Usually, images are processed for two reasons, first, for information extraction and second to improve their quality. To accomplish this, we need to pre-process these images; using techniques that help us obtain a more suitable image for the required application [4], [5], [6], [7].

There are several methods for noise elimination and edge detection [1], [2], [6], [8]. The processes mentioned above are considered to be opposite of each other because edge detection emphasizes the changes in the image tones while noise elimination minimizes these changes [7], [9].

Edges detection is one of the most widely used methods for image pre-processing since it is much faster than processing whole images from the start. In this way, total time execution is reduced dramatically.

Some of these methods are Sobel, Prewitt, Roberts, Morphological Gradient, Canny and others [2], [6],[8], [9], [10]. Some variants of the traditional methods include the use of neural networks [3], [7], genetic algorithms or hybrid systems [11], [12], [13], [14], [15], [16]. Edge detection can improve the results of different image processing systems. But the main question remains, how can we choose the best edge detector for a given problem?.

There are different metrics for edges evaluation [17], [18], [19], [20], [21]; all of them are based on finding the similarity between two images. The first image corresponds to the detected edges, and the second image is the reference image or ground truth (GT), which is considered the image with the ideal edges.

**Fig 1:**
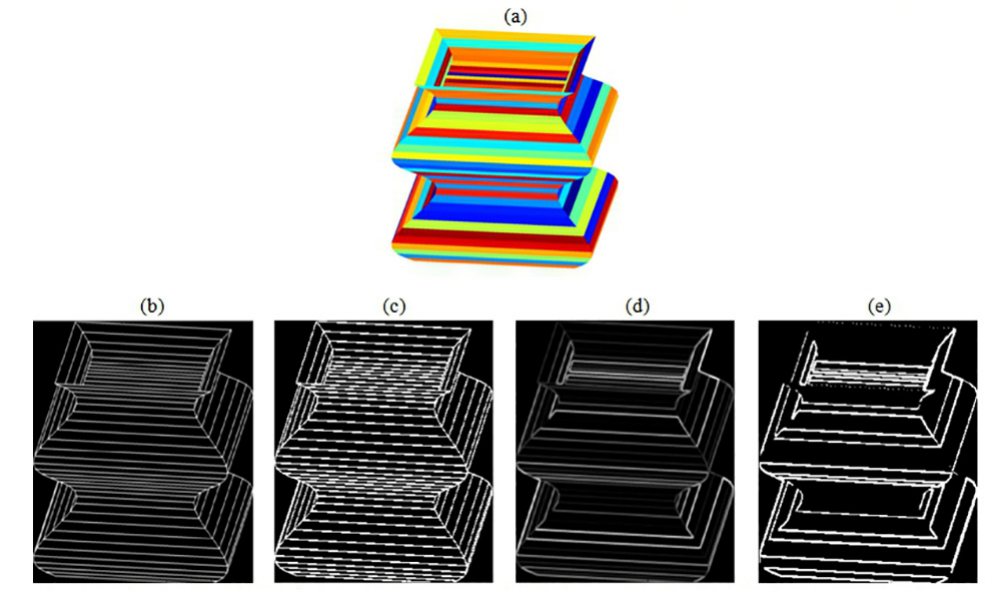
Binarization of edges images. (a) Color image, (b) GT image, (c) Binarized GT, image, (d) Edges image, (e) Binarized edges image.

All existing metrics include a previous step consisting on image binarization, but much information is lost and, as a result incomplete images will be evaluated. We show a color synthetic image in Fig 1(a), a wired synthetic image (GT) in Fig 1(b) and detected edges image in Fig 1(d). Images in Fig 1(b) and 1(d) are not binarized, while images in Fig 1(c) and 1(e) are binarized. The difference between images can be noticed easily, the loss of edges regions in binarized images is evident.

It has been shown that neural network systems for image processing get greater image recognition percentage when images used in training phase are processed with edges detection algorithms, specifically those improved using fuzzy inference systems [21][22].

After visual inspection, images from fuzzy edges detectors show more details of original images and more homogeneous background. Better feature extraction allows train neural networks with more relevant attributes and less noise in data sets, improving their performance.

While visual analysis is very useful, it is also qualitative and subjective. For a more objective analysis, it is mandatory to quantify performance. Up to now, we have only found edges detector evaluation methods which need to binarize images before processing, with the disadvantage that the evaluated image will not be the same as the one actually used by the system.

The main contribution of this paper consists in a proposed method to calculate a fuzzy index for the evaluation of the detected edges of an image represented by its fuzzy form (without binarization) [4], [8], [9], [10], [16], [19].

The proposed fuzzy index FI integrates parameters that other metrics use separately. As shown in Table 1.

**Table 1:** Comparison of metrics for edges detection evaluation.

	Parameters				
Metrics	Euclidean Distance	False Positive (FP)	True Positive (TP)	False Negative (FN)	Binarize
FOM	**✓**				**✓**
DC		**✓**	**✓**	**✓**	**✓**
JI		**✓**	**✓**	**✓**	**✓**
FI	**✓**	**✓**	**✓**	**✓**	

Since the proposed method was designed for fuzzy images, all the calculations were extended with fuzzy operators. Another significant contribution is the fuzzy extension of Euclidean distance [23] between two images; as part of the method for the calculation of an index to evaluate edge detection.

The paper is organized as follows: In a Section 2 we describe some metrics used to evaluate edge detection: The Euclidean distance [23], Pratt's Figure of Merit [20], Jaccard’s index and Dice’s coefficient [18]. In Section 3 the basic concepts of fuzzy sets operations, like intersection, bounded difference and magnitude of fuzzy set [5],[8] are described. In Section 4, we describe the proposed method, including fuzzy Euclidean distance and the algorithm to compute it.

Finally, we explain how parameters described in Section 3 are integrated, and an algorithm to compute fuzzy index (FI) is proposed. In Section 5, we show experiments and results made with different synthetic images and their comparison with traditional metrics. Section 6 is for our conclusions.

## Metrics for Edges Detection Evaluation

In this section, we describe some existing metrics for edge detection evaluation. These methods are key for designing the proposed FI.

### Euclidean distance between two images

The Euclidean distance between two points, as shown in Eq 1, is commonly used to find the similarity between two images. If one of the images is assigned as reference of correctly detected edges, the Euclidean distance can be a quality measure of the detected edges of another image.

$$Ed(A_{x_{1},y_{1}},B_{x_{2},y_{2}})\;=\;\sqrt{{\left(x_{2}-x_{1}\right)}^{2}+{\left(y_{2}-y_{1}\right)}^{2}}$$ (1)

**Fig 2:**
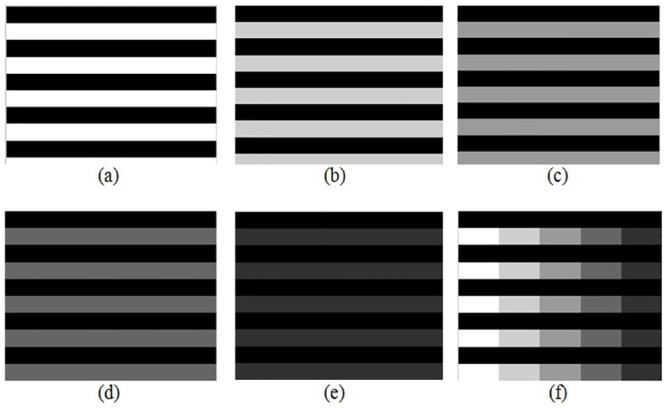
Euclidean distance matrices between images A and B. (a) Image A, (b) Image B, (c) Euclidean distance matrix between a_11_ and b_ij_, (d) Euclidean distance matrix between a_33_ and b_ij_.

Given two images A and B, the Euclidean distance between pixels can be calculated as follows:

- Fig 2(a) represent image A and Fig 2(b) represent image B.
- The image in Fig 2(c) shows the Euclidean distance from pixel *a*_*1*,*1*_ to all other pixels on B.
- A value of 0 (zero) corresponds to the Euclidean distance from pixel *a*_*1*,*1*_ to pixel *b*_*1*,*1*_ and increases as pixels move farther.
- The image in Fig 2(d) shows Euclidean distances from *a*_*3*,*3*_ to all other pixels on B.

### Pratt's figure of merit (FOM)

One of the most used metrics is the Pratt's Figure of merit Eq 2 [20]. Abdou and Pratt proposed this metric in 1978 [17].

$$FOM\;=\;\frac{1}{max(N_{i}{,N}_{d})}\sum_{i\;=\;1}^{N_{d}}\frac{1}{1+\propto d_{i}^{2}}$$ (2)

It uses Euclidean distance $d_{i}^{2}$ [23] to compare two images, the first is the image of reference edges *N*_*i*_, which is also called Ground truth (GT) and the second is the detected edges image *N*_*d*_. It multiplies a scale factor ∝ to the Euclidean distance calculated between the two images to penalize detected edges, this factor can vary or not even be used, and then it normalizes values and makes the sum of all calculations. Finally, it is multiplied by the inverse of the maximum amount of edge pixels between the two compared images.

It is noteworthy that these metrics binarize data before evaluating images; this means that evaluation is made over images that have lost information. These metrics return values between 0 and 1, where 0 would mean that there were no similarities found between detected image and reference image, and 1 meaning that great similarity was found, in other words, all pixels found in one image edges are detected in the same position as the other.

### Jaccard´s index (JI) and Dice´s coefficient (DC)

**Fig 3:**
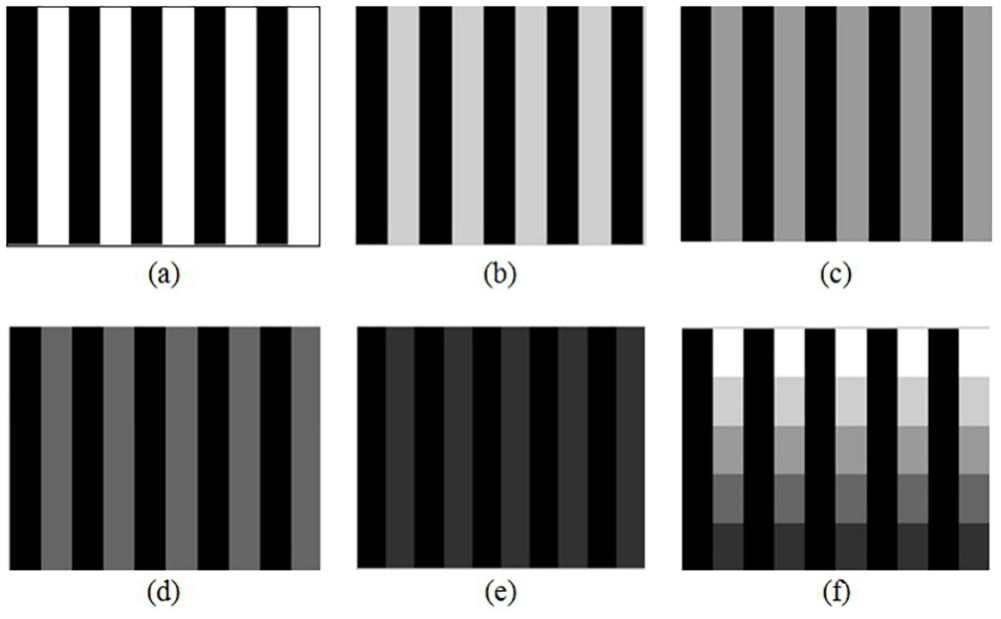
Results Set and Truth Set.

We also considered Jaccard´s index and Dice´s coefficient metrics [18], as shown below, which are based on rendering images with sets. Detected images are represented by set *Results Set* and reference images are represented by set *Truth Set* as is shown in Fig 3.

In *Results Set* we have: False Positive (FP) or “false alarms” (these are pixels marked as edges when they are not), and True Positive (TP) (which are truly edges); in *Truth Set* we have True Positive (TP) and False Negative (FN) (these are pixels marked as not edges, but they really are). These metrics are considered in the evaluation as false alarms (FP) and not detected edges (FN). The evaluation uses values between 0 and 1, where 0 means that there is no similarity between the images and 1 that the images are the same, but it also binarizes information before any evaluation is made.

Jaccard´s index (JI) and Dice´s coefficient (DC) use sets to represent images, as shown in Eqs 3 and 4 respectively. With the help of some set operations we are able to measure any detected edges, and these measures are some of the most applied.

$$JI\;=\;\frac{TP}{FP+TP+FN}$$ (3)

$$DC\;=\;2\left(\frac{TP}{\left(FP+TP\right)+(TP+FN)}\right)$$ (4)

### Fuzzy sets

Today we can find a variety of areas where fuzzy logic is applied [24], [25], [26], [27], [28], [29], [30], [31]. For example, control is one of the areas where it is most used, simulation, prediction, optimization, information systems, such as databases, pattern recognition [32], [33], [34], [35], [36], [37] computer vision, etc. Fuzzy logic is an alternative to traditional logic, which assigns a degree of membership to evaluate things such as human reasoning.

Fuzzy logic started in 1965 by Lotfi Zadeh, Professor at the University of Berkeley, mainly applied in control systems and complex industrial processes, working with information that is not accurate or with high inaccuracy [38].

There are concepts that do not have clear boundaries, this is why these fuzzy sets are associated to a linguistic value. Its membership function is defined in the range between 0 and 1, as shown in Eq 5.

A fuzzy set has been defined as shown in Eq 6, where *μ*_A_ is the membership function of variable x in the universe of discourse *U*. The membership function is the essence of fuzzy sets and operations between fuzzy sets are based on it.

$$\mu_{A}:x\to \left[0,1\right]$$ (5)

$$A\;=\;\left\{\left(x,\mu_{A}(x)\right)|\;x\;\in \;U\right\}$$ (6)

### Fuzzy sets operators

There are different operations between fuzzy sets, such as union, intersection, complement, etc. In this paper we use intersection, bounded difference and fuzzy scalar cardinality, these are explained below.

#### Intersection of fuzzy sets

Given sets A and B, intersection C is defined as Eq 7. For fuzzy sets Zadeh introduced fuzzy intersection defined in Eq 8 as the search for minimum of two fuzzy sets [38].

C = {x|x∈A and x∈B} (7)

$$\mu_{A\cap B\;=\;\text{min}\left\{\mu_{A}\left(x\right),\mu_{B}(x)\right\}}$$ (8)

#### Bounded difference of fuzzy sets

Given sets A and B, bounded difference C is defined as shown in Eq 9. Fuzzy bounded difference between A and B is defined in Eq 10 as the maximum of 0 and the difference between each membership values of A and B. This operator is not commutative, then the bounded difference between B and A is defined in Eq 11.

$$C\;=\;\left(A\;\theta \;B\right)\;\forall x\in \;\cup$$ (9)

$$\mu_{A\theta B\;=\;\text{max}\left\{0,\left\{\mu_{A}\left(x\right)-\mu_{B}\left(x\right)\right\}\right\}}$$ (10)

$$\mu_{B\theta A\;=\;\text{max}\left\{0,\left\{\mu_{B}\left(x\right)-\mu_{A}\left(x\right)\right\}\right\}}$$ (11)

#### Magnitude of fuzzy sets

The magnitude of a fuzzy set can be calculated in different ways, one of which is the scalar cardinality defined as the sum of all the fuzzy values of each of the elements of the fuzzy set represented by Eq 12.

$$\left|A\right|\;=\;\sum_{x\epsilon X}{\;\mu }_{A}\left(x\right)$$ (12)

## The Proposed Method

In the proposed method we introduce the calculation of the following fuzzy operations between a fuzzy reference image of edges and a fuzzy detected image: The fuzzy Euclidean distance (FD), the fuzzy true positive (FTP), the fuzzy false positive (FFP) and the fuzzy false negative (FFN).

**Fig 4:**
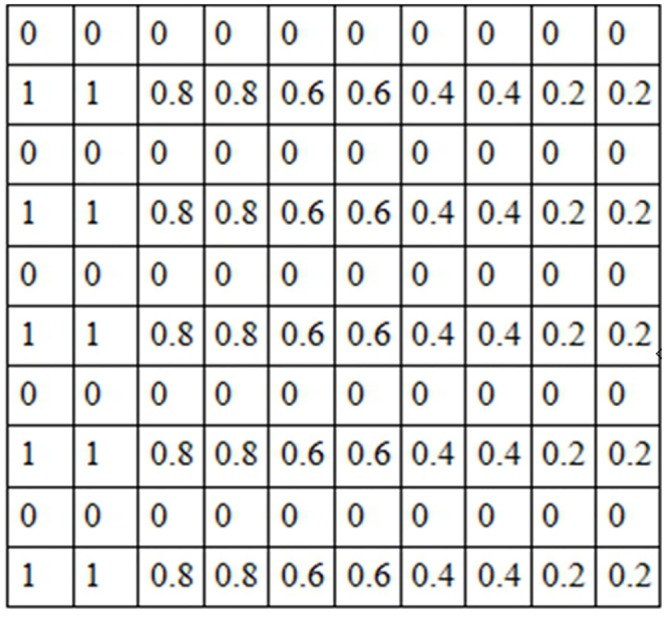
Diagram for the index calculation.

The method integrates all of them in a single operator named fuzzy index (FI). A diagram of the process is shown in Fig 4.

### The fuzzy Euclidean distance

Given two images A and B, the fuzzy Euclidean distance between each pixel a_*i*,*j*_ in A and all the pixels *b*_*i*,*j*_ in B can be calculated with Eq 1 as described in section 2.1.

**Fig 5:**
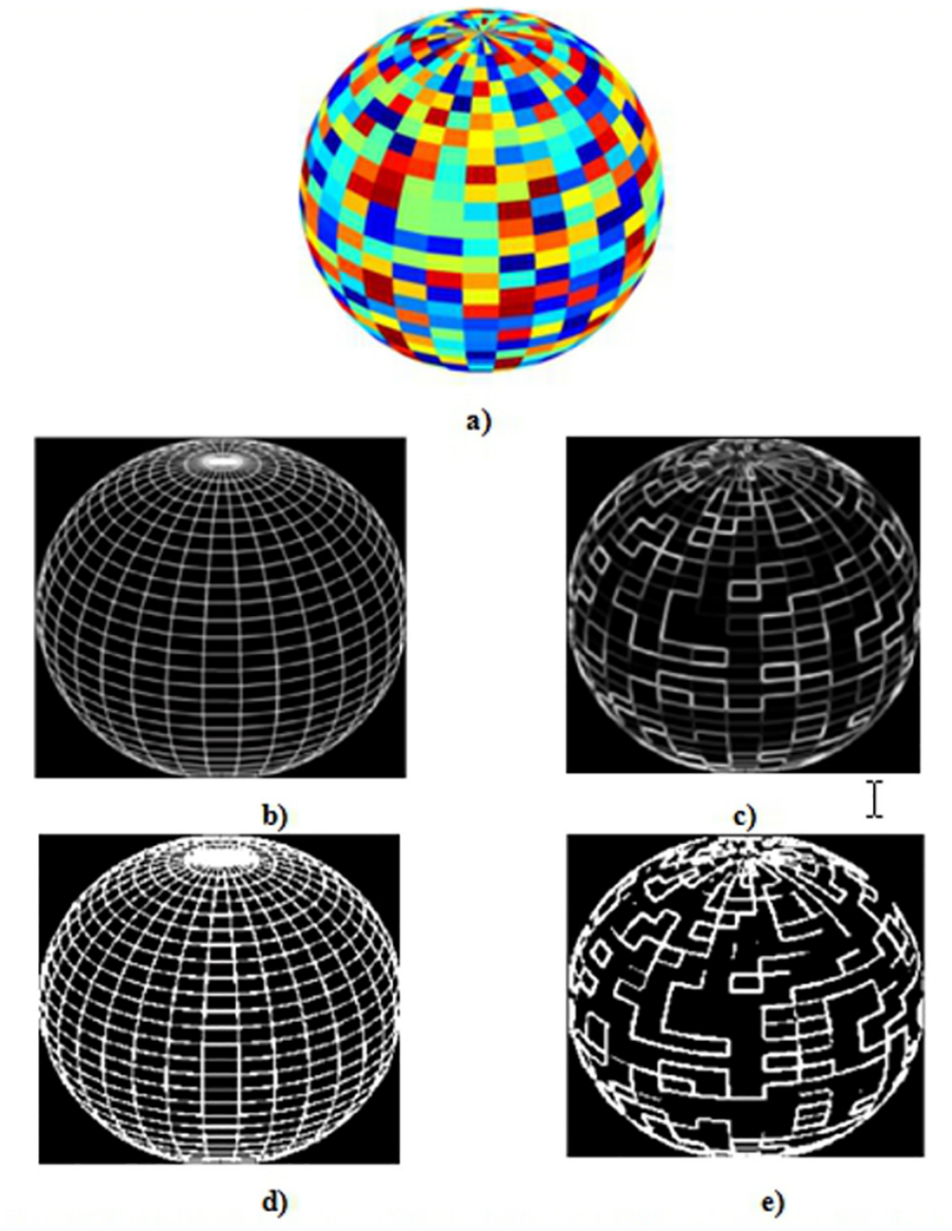
Euclidean distance matrices between images A and B. (a) Image A, (b) Image B, (c) Fuzzy Euclidean distance matrix between a_11_ and b_ij_, (d) Fuzzy Euclidean distance matrix between a_33_ and b_ij_.

Once the Euclidean distance matrix D was calculated, the fuzzy Euclidean distance matrix FD can be obtained with some fuzzy membership function. In our method, we propose a triangular membership function as shown in Fig 5.

Once the Euclidean distance matrix D was obtained, the fuzzy Euclidean distance matrix FD can be calculated with some fuzzy membership function. In our method we propose a triangular membership function.

### Fuzzy synthetic images

**Fig 6:**
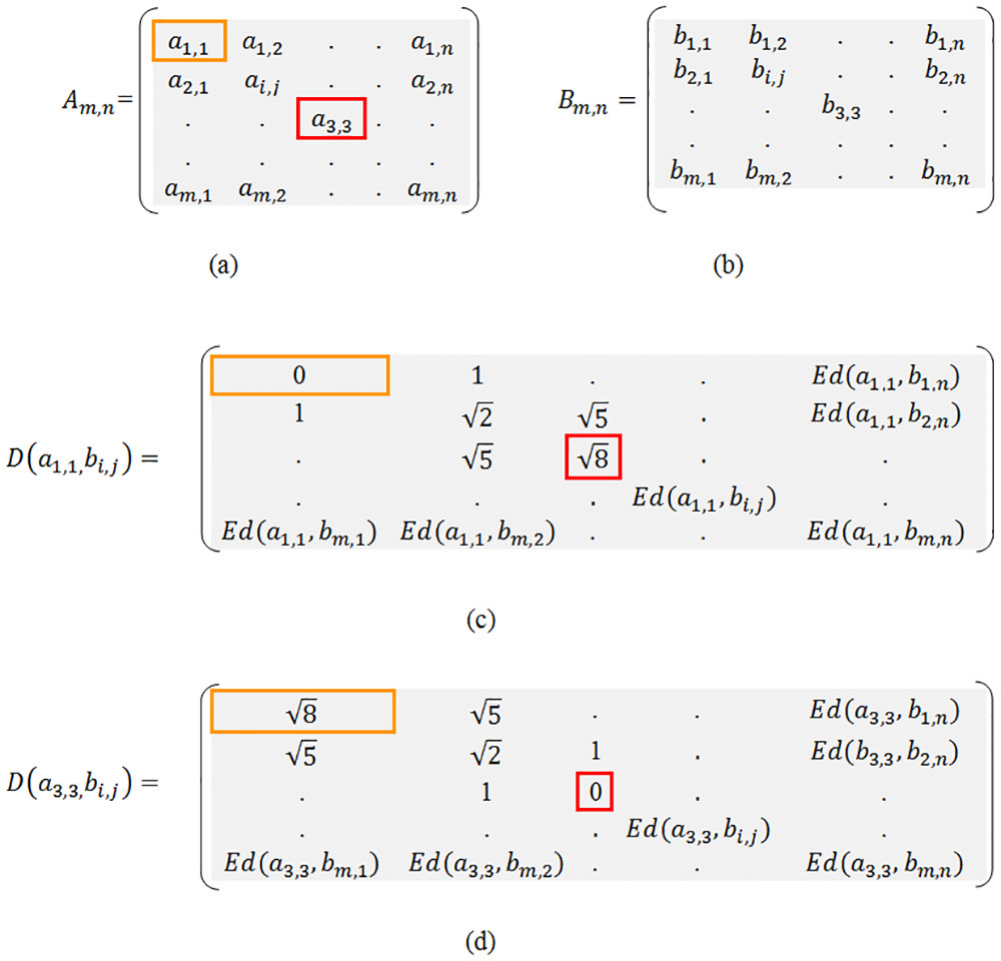
Image in gray map.

**Fig 7:**
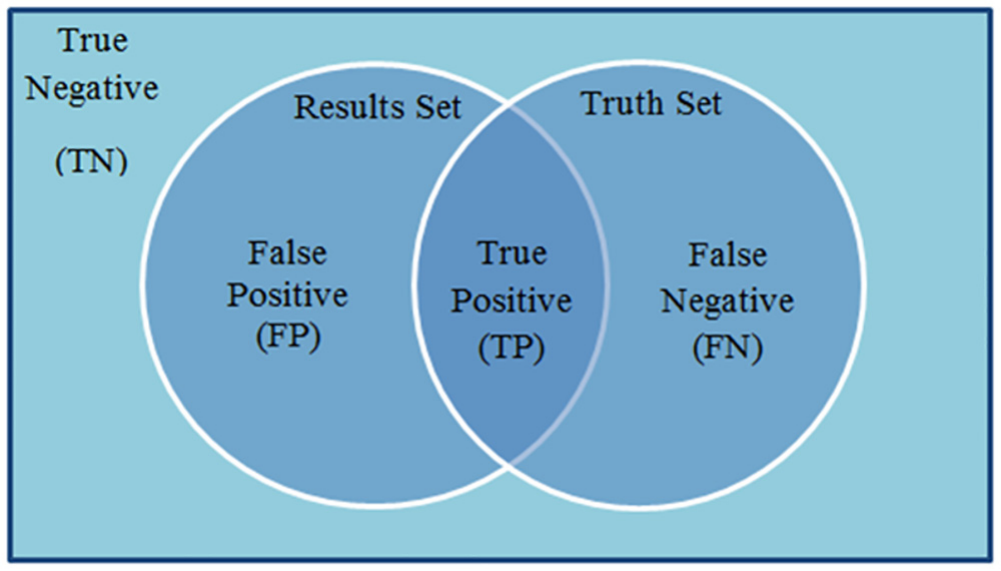
Pixels matrix in gray map between 0 and 255.

Images can be stored in matrices where each pixel is represented with values in the interval [0,255], as we can see in Figs 6 and 7. Particularly in binarized edges images, components with values of 1 represent pixels classified as edges, and components with values of 0 represent non edge pixels.

For fuzzy images approach, in edges images, all pixels are classified as edges with different membership values.

**Fig 8:**
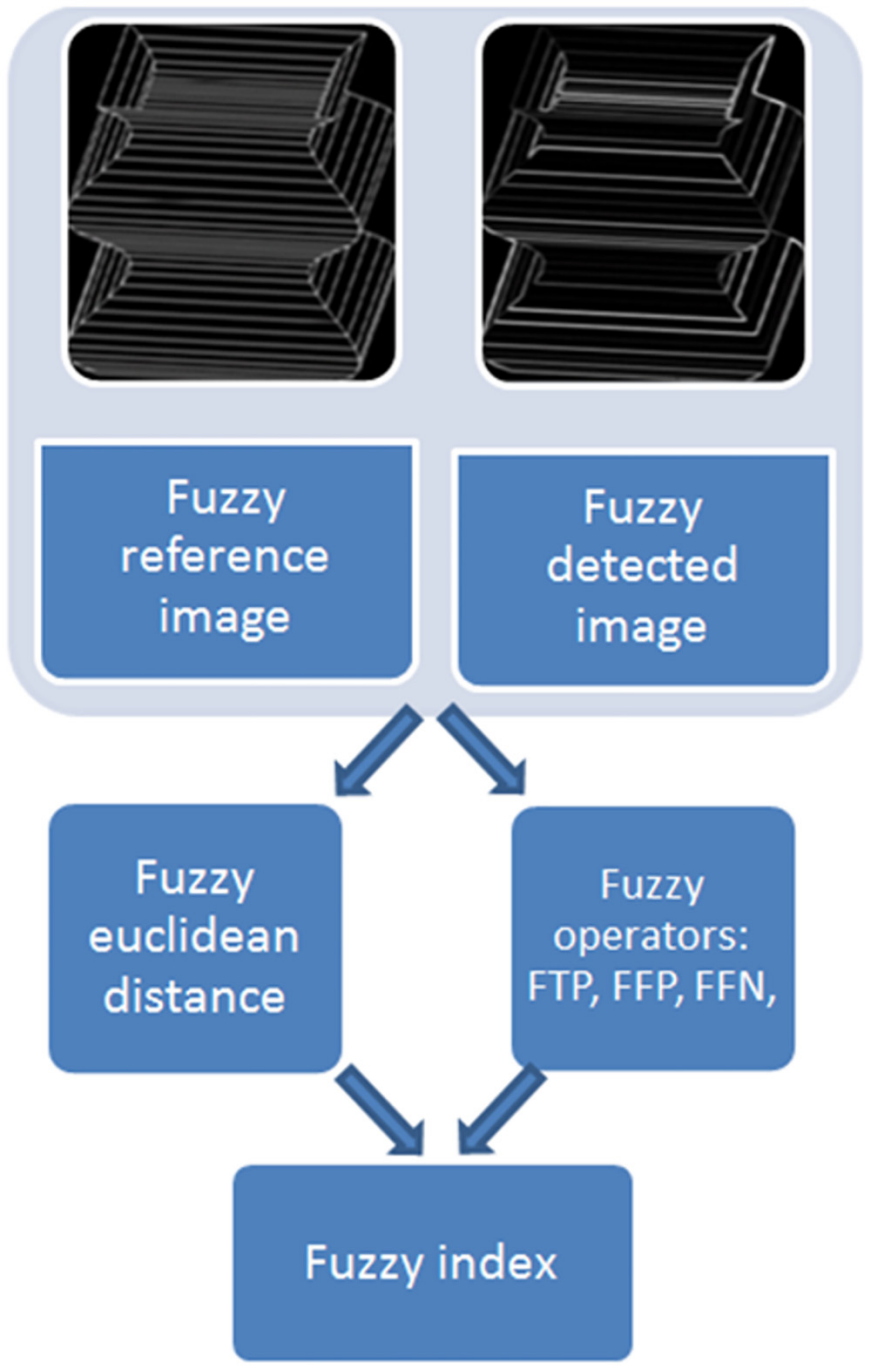
Pixels matrix after fuzzification with a triangular membership function

The fuzzy values using the triangular membership functions in Eq 13 according to the degrees of membership rightfully assigned are shown in Fig 8.

$${\;\mu }_{A}\left(x\right)\;=\;\frac{x}{max(A)}$$ (13)

**Fig 9:**
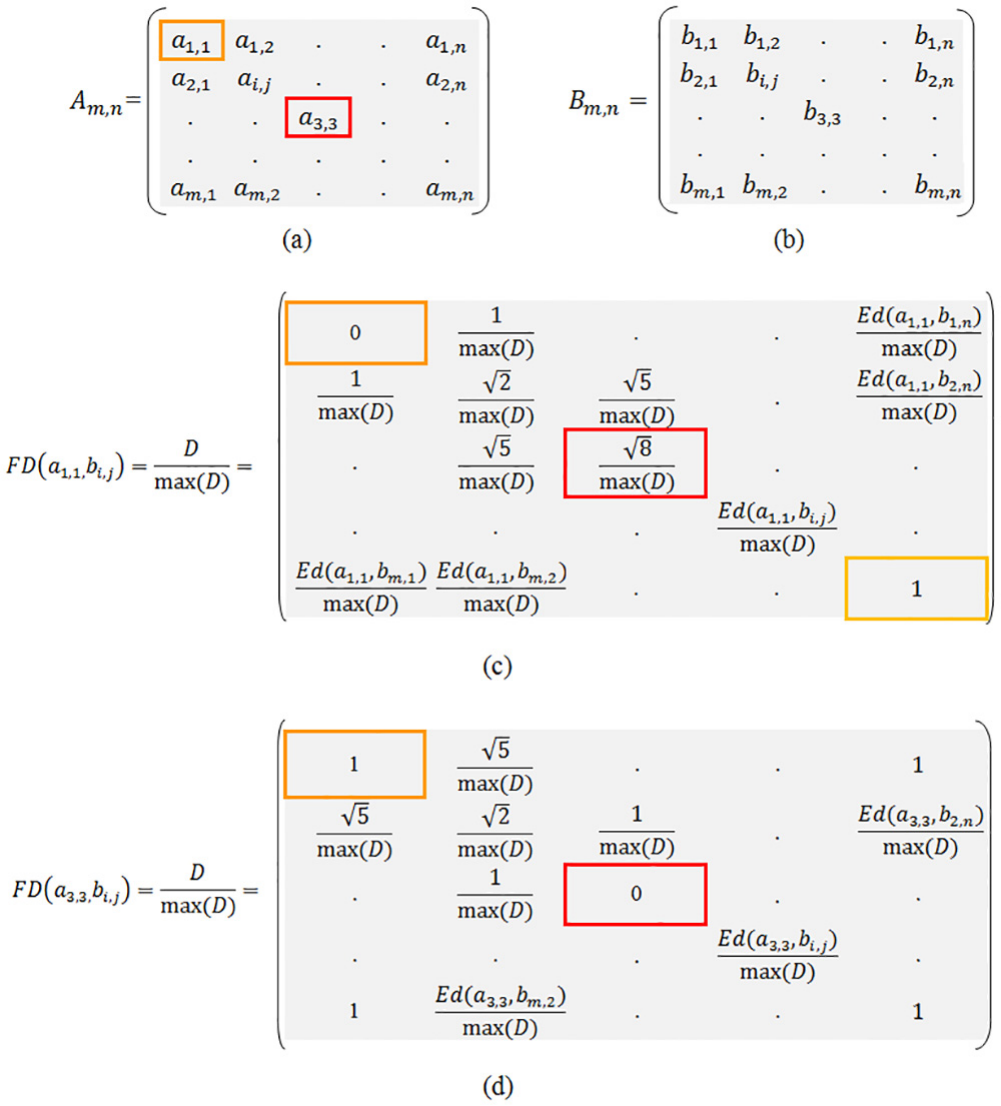
Synthetic grid images. (a)Synthetic image with values 0and 1, (b) Synthetic image with values 0 and 0.8, (c) Synthetic image with values 0 and 0.6, (d) Synthetic image with values 0 and 0.4, (e) Synthetic image with values 0and 0.2, (f) Synthetic image with values 0, 0.2, 0.4, 0.6, 0.8 and 1.

**Fig 10:**
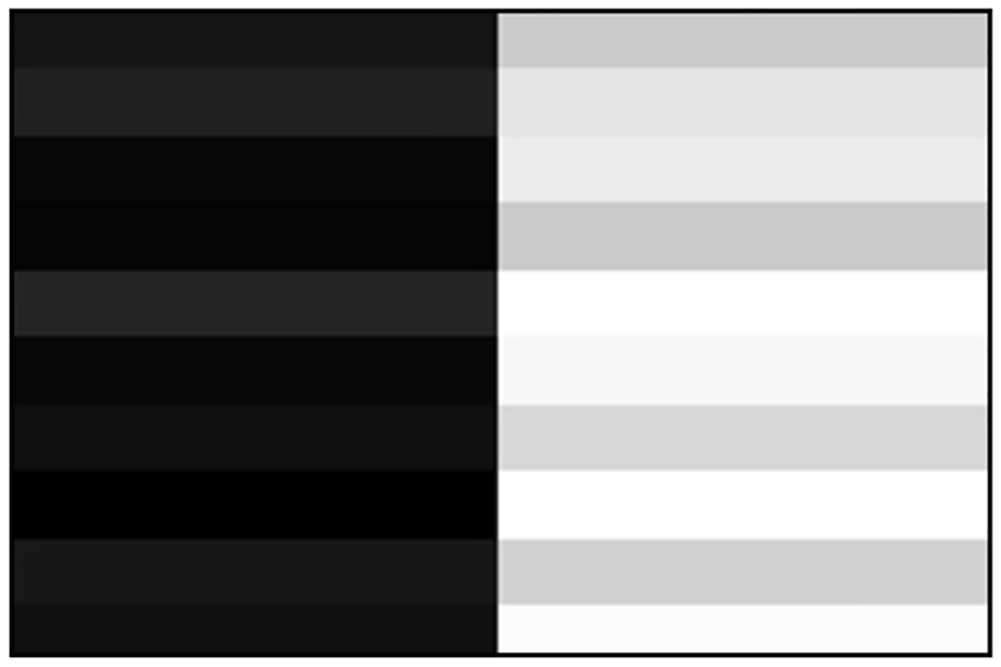
Synthetic horizontal images. (a) Synthetic image. (a) Synthetic image with values 0and 1, (b) Synthetic image with values 0 and 0.8, (c) Synthetic image with values 0 and 0.6, (d) Synthetic image with values 0 and 0.4, (e) Synthetic image with values 0and 0.2, (f) Synthetic image with values 0, 0.2, 0.4, 0.6, 0.8 and 1.

**Fig 11:**
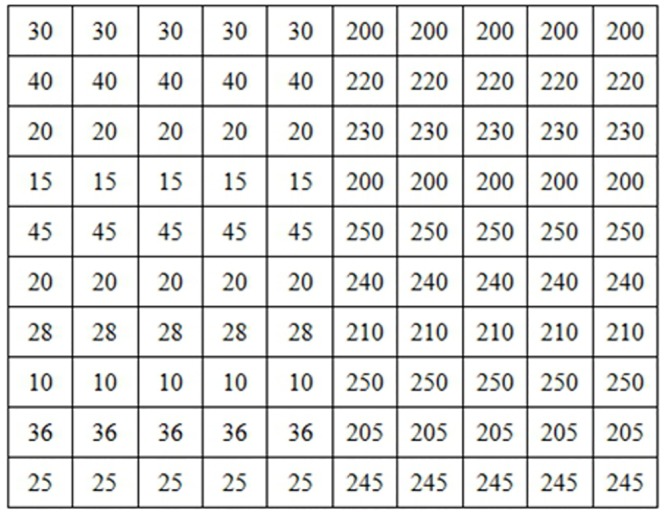
Synthetic vertical images. (a)Synthetic image with values 0and 1, (b) Synthetic image with values 0 and 0.8, (c) Synthetic image with values 0 and 0.6, (d) Synthetic image with values 0 and 0.4, (e) Synthetic image with values 0and 0.2, (f) Synthetic image with values 0, 0.2, 0.4, 0.6, 0.8 and 1.

In order to test that our proposed method finds the correct index for a given pair of images, synthetic images were generated as shown in the Figs 9,10 and 11 in order to control the amount of edges, and also to manipulate gray tones of pixels and to show their differences.

**Fig 12:**
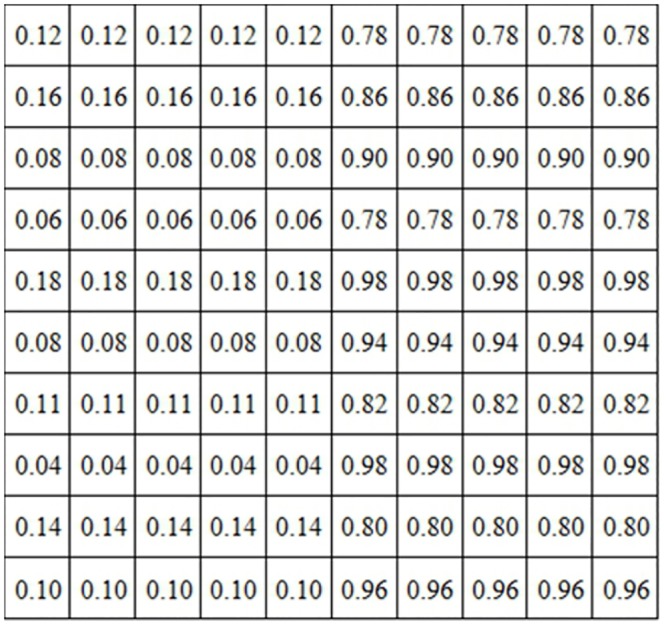
Example for the matrix to generate the Fig 10(f).

Each image was a 10x10 matrix with values in the interval [0,1], where 0 indicates an absence of color or black and 1 means white, e.g. image in Fig 10(f) can be generated with matrix shown in Fig 12.

### Fuzzy sets operators for fuzzy images

Fuzzy sets operators between two fuzzy images can be defined according to the theory of fuzzy sets and the definitions in section 2.3.

#### Fuzzy true positive (*FTP*) between two fuzzy images

The set of true positive pixels between fuzzy images *A* and *B* is defined as the intersection of *A* and *B*. Then, *FTP* can be calculated using Eq 7.

#### Fuzzy false positive (*FFP*) between two fuzzy images

The set of false positive pixels between fuzzy images *A* and *B* is defined as the bounded difference between *A* and *B*. Then, *FFP* can be calculated using Eq 9.

#### Fuzzy false negative (*FFN*) between two fuzzy images

The set of false negative pixels between fuzzy images *A* and *B* is defined as the bounded difference between *B* and *A*. Then, *FFN* can be calculated using Eq 10.

#### Magnitude of a fuzzy image

The magnitude of a fuzzy image can be defined as the scalar cardinality *|A|* of a fuzzy image using Eq 11.

### Calculation of the fuzzy index

Synthetic fuzzy images used in our proposal are matrices calculated by Eqs 14 and 15. The fuzzyfication of the images were made with triangular membership functions as described in Eq 13.

$$N_{d}\;=\;\left\{d_{11},d_{12},d_{13},\ldots d_{mn}\right\}\;=\;{\;\mu }_{DI}\left(x\right)\;=\;\frac{x}{max(DI)}$$ (14)

$$N_{i}\;=\;\left\{i_{11},i_{12},i_{13},\ldots i_{mn}\right\}\;=\;{\;\mu }_{II}\left(x\right)\;=\;\frac{x}{max(II)}$$ (15)

Where *DI* are the edges detected image, *II* are the reference image (ground truth), *N*_*d*_ are the fuzzy detected image and *N*_*i*_ are the fuzzy reference edge image (Ground Truth or GT).

In our test, we used synthetic images stored in matrices *N*_*i*_ and *N*_*d*_ represented in their fuzzy form. Since images were already fuzzy, we started calculating fuzzy Euclidean distance. Then, we integrate FTP, FFP, FFN and Euclidean distance (FD) for the most similar pixels to obtain FI using Eq 16.

FI = 1m ∙n∙max⁡Ni,NdFTP∑1≤ i ≤ m1≤j≤n 11+FDi,j-FFP-FFN (16)

### Algorithms

The algorithm for calculating the FI is divided into two phases: one that calculated the fuzzy Euclidean distance matrix FD and the second that calculated the FI. Then algorithms are shown in pseudo code as follows.

#### Algorithm to compute the fuzzy Euclidean distance FD of the most similar pixels between *N*_*i*_ and *N*_*d*_

This algorithm takes the images ***N***_***i***_ and ***N***_***d***_ to calculate the fuzzy Euclidean distance of each pixel on ***N***_***i***_ to the most similar pixel on ***N***_***d***_.

**Input**: Fuzzy images *N*_*d*_ = {*d*_11_,*d*_12_,*d*_13_, …*d*_*mn*_} and *N*_*i*_ = {*i*_11_,*i*_12_,*i*_13_, …*i*_*mn*_}

**Output**: Fuzzy Euclidean distance matrix *FD*(*N*_*i*_,*N*_*d*_)

1. For each pixel in *N*_*i*_(*x*_1_,*y*_1_)do steps 2 to 4
2. Calculate the difference between the pixel *N*_*i*_(*x*_1_,*y*_1_) and all pixels on $N_{d}$ with
  $$dif\left(x_{1},\;y_{1}\right)\;=\;|N_{i}\left(x_{1},\;y_{1}\right)-N_{d}\left(i,\;j\right)|$$
3. Found the most similar pixels to *N*_*i*_(*x*_1_,*y*_1_) in *N*_*d*_ as those with the minimum value in *dif*(*x*_1_,*y*_1_)
4. For each most similar pixel in *N*_*d*_(*x*_2_,*y*_2_) do steps 5 and 6
5. Calculate the Euclidean distance between *N*_*i*_(*x*_1_,*y*_1_) and *N*_*d*_(*x*_2_,*y*_2_) *with*
  $$D\left(x1,y1\right)\;=\;\sqrt{{\left(x_{2}-x_{1}\right)}^{2}+{\left(y_{2}-y_{1}\right)}^{2}}$$
6. Replace *D*(*x*1,*y*1) if a nearest most similar pixel is founded
7. Calculate the maximum Euclidean distance between two elements in D with
  $$maxD\;=\;\sqrt{{\left(m-1\right)}^{2}+{\left(n-1\right)}^{2}}$$
8. Calculate the Fuzzy Euclidean distance matrix with
  $$FD\left(N_{i},N_{d}\right)\;=\;\left(\frac{D\left(i,j\right)}{maxD}\right)$$

#### Algorithm to compute the proposed fuzzy index

This algorithm takes the images ***N***_***i***_ and ***N***_***d***_ and ***FD*** to calculate the fuzzy index ***FI***

**Input**: Fuzzy images *N*_*d*_ = {*d*_11_,*d*_12_,*d*_13_, …*d*_*mn*_}, *N*_*i*_ = {*i*_11_,*i*_12_,*i*_13_, …*i*_*mn*_}, and the fuzzy Euclidean distance matrix *FD*_m,n_(*N*_*i*_,*N*_*d*_).

**Output**: Fuzzy index *FI*.

1. Calculate the scalar cardinality *N*_*i*_ and *N*_*d*_ with
  Nd = ∑0≤ i ≤ m1≤j≤n Ndi,j
  Ni = ∑0≤ i ≤ m1≤j≤n Nii,j
2. Calculate *FTP* as the fuzzy intersection of *N*_*i*_ and *N*_*d*_ with
  $$FTP\;=\;min(N_{i}\left(i,j\right),N_{d}\left(i,j\right))$$
3. Calculate the scalar cardinality of *FTP* with
  FTP = ∑0≤ i ≤ m1≤j≤n FTPi,j
4. Calculate FFP as the bounded difference between *N*_*i*_ and *N*_*d*_ with
  $$FFP\;=\;max\{0,\left\{\;N_{i}\left(i,j\right)-N_{d}\left(i,j\right)\right\}\}$$
5. Calculate the scalar cardinality of *FFP* with
  FFP = ∑0≤ i ≤ m1≤j≤n FFPi,j
6. Calculate *FFN* as the bounded difference between *N*_*d*_ and *N*_*i*_
  $$FFN\;=\;max\{0,\left\{\;N_{d}\left(i,j\right)-N_{i}\left(i,j\right)\right\}\}$$
7. Calculate the scalar cardinality of *FFN*
  FFN = ∑0≤ i ≤ m1≤j≤n FFNi,j
8. Calculate fuzzy index *FI* with
  FI = 1m∙n∙max⁡Ni,NdFTP∑1≤ i ≤ m1≤j≤n 11+FDi,j-FFP-FFN

## Experiments and Results

In this section we compare the results of the calculation of the fuzzy sets operators proposed in this paper, with non fuzzy sets operators. The calculations were made with all the images shown in Figs 9, 10 and 11, and all of them yield similar results. In Table 2, are shown the results of fuzzy and non fuzzy sets operators for some comparisons between images in Fig 10 (S1 Dataset).

**Table 2:** Results of the comparison of *N*_*i*_ and *N*_*d*_ of Fig 6 with non fuzzy sets and fuzzy sets operators.

#	N_i_	N_d_	%TP	%FN	%FP	%FTP	%FFN	%FFP
1	Fig 10(a)	Fig 10(a)	100.00	0.00	0.00	**100.00**	**0.00**	**0.00**
2	Fig 10(a)	Fig 10(b)	100.00	0.00	0.00	**83.33**	**0.00**	**16.67**
3	Fig 10(a)	Fig 10(c)	100.00	0.00	0.00	**62.50**	**0.00**	**37.50**
4	Fig 10(a)	Fig 10(d)	100.00	0.00	0.00	**41.67**	**0.00**	**58.33**
5	Fig 10(a)	Fig 10(e)	100.00	0.00	0.00	**20.83**	**0.00**	**79.17**
6	Fig 10(a)	Fig 10(f)	80.00	20.00	0.00	**61.67**	**0.83**	**38.33**
7	Fig 10(b)	Fig 10(a)	100.00	0.00	0.00	**83.33**	**16.67**	**0.00**
8	Fig 10(b)	Fig 10(b)	100.00	0.00	0.00	**100.00**	**0.00**	**0.00**
9	Fig 10(b)	Fig 10(c)	100.00	0.00	0.00	**75.00**	**0.00**	**25.00**
10	Fig 10(b)	Fig 10(d)	100.00	0.00	0.00	**50.00**	**0.00**	**50.00**
11	Fig 10(b)	Fig 10(e)	100.00	0.00	0.00	**25.00**	**0.00**	**75.00**
12	Fig 10(b)	Fig 10(f)	80.00	20.00	0.00	**70.00**	**5.00**	**30.00**
13	Fig 10(c)	Fig 10(a)	100.00	0.00	0.00	**62.50**	**37.50**	**0.00**
14	Fig 10(c)	Fig 10(b)	100.00	0.00	0.00	**75.00**	**25.00**	**0.00**
15	Fig 10(c)	Fig 10(c)	100.00	0.00	0.00	**100.00**	**0.00**	**0.00**
16	Fig 10(c)	Fig 10(d)	100.00	0.00	0.00	**66.67**	**0.00**	**33.33**
17	Fig 10(c)	Fig 10(e)	100.00	0.00	0.00	**33.33**	**0.00**	**66.67**
18	Fig 10(c)	Fig 10(f)	80.00	20.00	0.00	**80.00**	**20.00**	**20.00**
19	Fig 10(d)	Fig 10(a)	100.00	0.00	0.00	**41.67**	**58.33**	**0.00**
20	Fig 10(d)	Fig 10(b)	100.00	0.00	0.00	**50.00**	**50.00**	**0.00**
21	Fig 10(d)	Fig 10(c)	100.00	0.00	0.00	**66.67**	**33.33**	**0.00**
22	Fig 10(d)	Fig 10(d)	100.00	0.00	0.00	**100.00**	**0.00**	**0.00**
23	Fig 10(d)	Fig 10(e)	100.00	0.00	0.00	**50.00**	**0.00**	**50.00**
24	Fig 10(d)	Fig 10(f)	80.00	20.00	0.00	**60.00**	**40.00**	**6.67**
25	Fig 10(e)	Fig 10(a)	100.00	0.00	0.00	**20.83**	**79.17**	**0.00**
26	Fig 10(e)	Fig 10(b)	100.00	0.00	0.00	**25.00**	**75.00**	**0.00**
27	Fig 10(e)	Fig 10(c)	100.00	0.00	0.00	**33.33**	**66.67**	**0.00**
28	Fig 10(e)	Fig 10(d)	100.00	0.00	0.00	**50.00**	**50.00**	**0.00**
29	Fig 10(e)	Fig 10(e)	100.00	0.00	0.00	**100.00**	**0.00**	**0.00**
30	Fig 10(e)	Fig 10(f)	80.00	20.00	0.00	**33.33**	**66.67**	**0.00**
31	Fig 10(f)	Fig 10(a)	80.00	0.00	20.00	**61.67**	**38.33**	**0.83**
32	Fig 10(f)	Fig 10(b)	80.00	0.00	20.00	**70.00**	**30.00**	**5.00**
33	Fig 10(f)	Fig 10(c)	80.00	0.00	20.00	**80.00**	**20.00**	**20.00**
34	Fig 10(f)	Fig 10(d)	80.00	0.00	20.00	**60.00**	**6.67**	**40.00**
35	Fig 10(f)	Fig 10(e)	80.00	0.00	20.00	**33.33**	**0.00**	**66.67**
36	Fig 10(f)	Fig 10(f)	100.00	0.00	0.00	**100.00**	**0.00**	**0.00**

The first observation was the value for all operators when two identical images were compared. For all operators, the similarity between the two images is 100% as expected. When we observe images in Fig 10(a) and 10(f), it is possible to note great differences. In this comparison results, we can observe that always the fuzzy operators found less true positives, more false positives and more false negatives that the non fuzzy operators. This results mean that the non fuzzy operators do not evaluate the image as we see it, because the binarization force the values to the extremes of the interval [0,1]. The fuzzy operators instead, evaluate de image as we see it, with all the details, because is not binarized.

Once we observe the results of fuzzy operators above, we compute the FI using algorithms described in section 4.5, and results are shown in Table 3. The indices are in the interval [0,1], where 1 means that images compared are identical.

**Table 3:** Results of the comparison of the images *N*_*i*_ and *N*_*d*_ on Fig 6 with the proposed index FI and non fuzzy metrics.

#	N_i_	N_d_	JI	DC	PFOM	FI
1	Fig 10(a)	Fig 10(a)	1.0000	1.0000	1.0000	**1.0000**
2	Fig 10(a)	Fig 10(b)	1.0000	1.0000	1.0000	**0.8317**
3	Fig 10(a)	Fig 10(c)	1.0000	1.0000	1.0000	**0.6213**
4	Fig 10(a)	Fig 10(d)	1.0000	1.0000	1.0000	**0.4108**
5	Fig 10(a)	Fig 10(e)	1.0000	1.0000	1.0000	**0.2004**
6	Fig 10(a)	Fig 10(f)	0.8000	0.8889	0.8000	**0.5549**
7	Fig 10(b)	Fig 10(a)	1.0000	1.0000	1.0000	**0.7416**
8	Fig 10(b)	Fig 10(b)	1.0000	1.0000	1.0000	**1.0000**
9	Fig 10(b)	Fig 10(c)	1.0000	1.0000	1.0000	**0.7475**
10	Fig 10(b)	Fig 10(d)	1.0000	1.0000	1.0000	**0.4950**
11	Fig 10(b)	Fig 10(e)	1.0000	1.0000	1.0000	**0.2425**
12	Fig 10(b)	Fig 10(f)	0.8000	0.8889	0.8000	**0.6458**
13	Fig 10(c)	Fig 10(a)	1.0000	1.0000	1.0000	**0.5537**
14	Fig 10(c)	Fig 10(b)	1.0000	1.0000	1.0000	**0.7475**
15	Fig 10(c)	Fig 10(c)	1.0000	1.0000	1.0000	**1.0000**
16	Fig 10(c)	Fig 10(d)	1.0000	1.0000	1.0000	**0.6633**
17	Fig 10(c)	Fig 10(e)	1.0000	1.0000	1.0000	**0.3267**
18	Fig 10(c)	Fig 10(f)	0.8000	0.8889	0.8000	**0.7449**
19	Fig 10(d)	Fig 10(a)	1.0000	1.0000	1.0000	**0.3987**
20	Fig 10(d)	Fig 10(b)	1.0000	1.0000	1.0000	**0.4950**
21	Fig 10(d)	Fig 10(c)	1.0000	1.0000	1.0000	**0.6633**
22	Fig 10(d)	Fig 10(d)	1.0000	1.0000	1.0000	**1.0000**
23	Fig 10(d)	Fig 10(e)	1.0000	1.0000	1.0000	**0.4950**
24	Fig 10(d)	Fig 10(f)	0.8000	0.8889	0.8000	**0.5518**
25	Fig 10(e)	Fig 10(a)	1.0000	1.0000	1.0000	**0.1928**
26	Fig 10(e)	Fig 10(b)	1.0000	1.0000	1.0000	**0.2334**
27	Fig 10(e)	Fig 10(c)	1.0000	1.0000	1.0000	**0.3145**
28	Fig 10(e)	Fig 10(d)	1.0000	1.0000	1.0000	**0.4950**
29	Fig 10(e)	Fig 10(e)	1.0000	1.0000	1.0000	**1.0000**
30	Fig 10(e)	Fig 10(f)	0.8000	0.8889	0.8000	**0.2936**
31	Fig 10(f)	Fig 10(a)	0.8000	0.8889	0.9592	**0.5763**
32	Fig 10(f)	Fig 10(b)	0.8000	0.8889	0.9592	**0.6914**
33	Fig 10(f)	Fig 10(c)	0.8000	0.8889	0.9592	**0.7902**
34	Fig 10(f)	Fig 10(d)	0.8000	0.8889	0.9592	**0.5953**
35	Fig 10(f)	Fig 10(e)	0.8000	0.8889	0.9592	**0.3267**
36	Fig 10(f)	Fig 10(f)	1.0000	1.0000	1.0000	**1.0000**

Once again we can observe that the binarization of the images made before the calculation of FOM, JI and DC, forces the comparison results. For non fuzzy operators, we can observe 100% in similitude for images evidently different.

**Fig 13:**
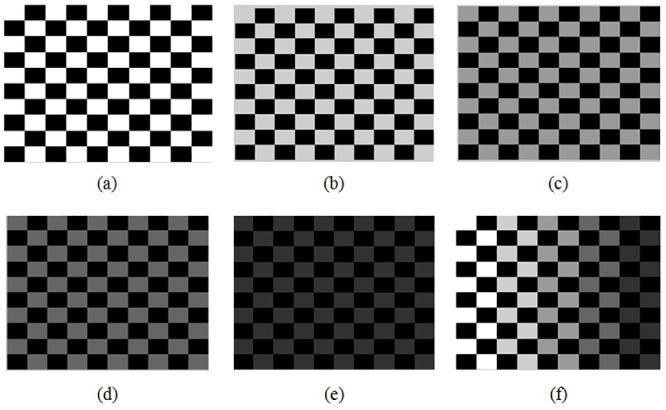
Reference and detected edges images of a sphere. a) Color image. b) Reference edges image without binarization, c) Detected edges image without binarization, d) Reference edges image after binarization, e) Detected edges image after binarization.

Synthetic images can be generated using some computer program, as the sphere shown in Fig 13. This image was generated using the function sphere of Matlab [39], this is an image of 200x200 (S2 Dataset).

The results on Table 4 shown that the non fuzzy indices FOM, JI and DC are identical, because the result is 1. But we can observe the images compared and found many differences. FI index calculates a very low similitude between the compared images, because of the few pixels correctly classified as edges, calculated as FTP. The non fuzzy operator TP instead, calculated a 100% of similitude between the images.

**Table 4:** Results of the comparison of the picture *N*_*i*_ and *N*_*d*_.

N_i_	N_d_	%TP	%FN	%FP	%FTP	%FFN	%FFP	JI	DC	PFOM	FI
Fig 13(b)	Fig 13(c)	44.76	55.23	30.56	15.89	74.09	84.10	0.3428	0.5106	0.6957	**0.1588**

With the fuzzy index proposed, we can compare two fuzzy images with high precision. Then if one of the images is a ground truth of edges image, we can evaluate the correct edges detection in fuzzy images.

## Conclusions

For a first conclusion of great importance, if the evaluation of an image without binarizing is carried out with more information about the image, you can have more pixels to work with.

The edge images without binarization used in classification systems allow us to achieve better recognition rates than the use of binarized images. Then the ability to evaluate the edges images in their fuzzy form can be used to preprocess data sets for optimal results.

Another important point is that we can use the simplest form of fuzzy logic to implement the evolution of images. We can notice in the results obtained that there is a great difference between the estimates obtained with traditional metrics because the FI we can give an index according to their gray tone and not simply dismiss it through a specified threshold.

These results show us that fuzzy logic is a good alternative in image processing and applied in many areas where it is extremely important edge detection.

The proposed fuzzy index is highly recommended, given the results. This fuzzy index includes parameters that other indices do not consider in their calculations, and allows us to compare two images represented as fuzzy sets.

Only synthetic images were used to calibrate proposed index FI, with very good results. Next step is to use it on real images. We are currently optimizing a fuzzy border detector to generate reference images that will help us evaluate border images given by our proposed index.

## Supporting Information

S1 Dataset Matrices used for the tests that show Tables 2 and 3. (ZIP)

S2 Dataset Images used for the tests that show Table 4. (ZIP)
