# Supplementary figures and images for: Fuzzy Index to Evaluate Edge Detection in Digital Images
Source: PLoS One. 2015 Jun 26;10(6):e0131161. doi: 10.1371/journal.pone.0131161 (PMC4483257; doi:10.1371/journal.pone.0131161)

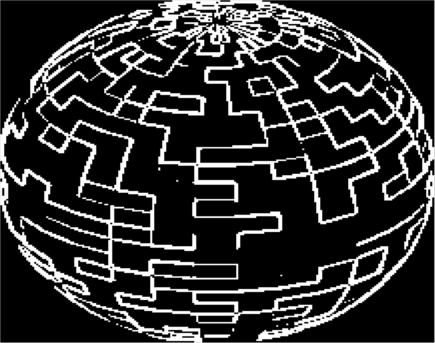

Supplement: S2 Dataset — (ZIP) [file pone.0131161.s002.zip › bin_detected_edges_sphere.png]

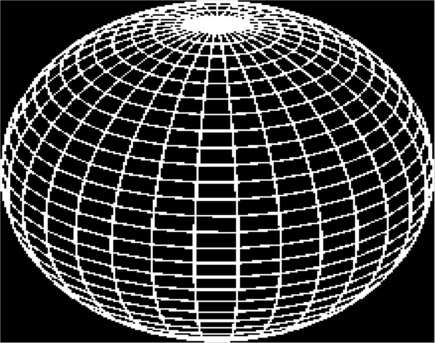

Supplement: S2 Dataset — (ZIP) [file pone.0131161.s002.zip › bin_gt_edges_sphere.png]

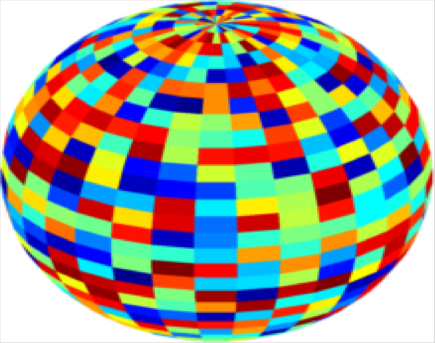

Supplement: S2 Dataset — (ZIP) [file pone.0131161.s002.zip › color_sphere.png]

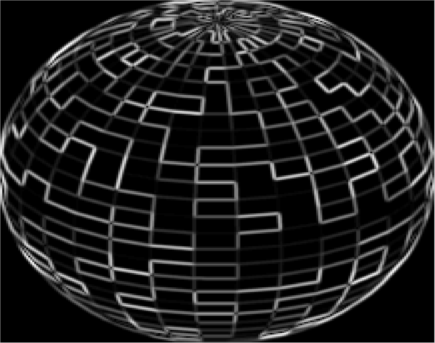

Supplement: S2 Dataset — (ZIP) [file pone.0131161.s002.zip › detected_edges_sphere.png]

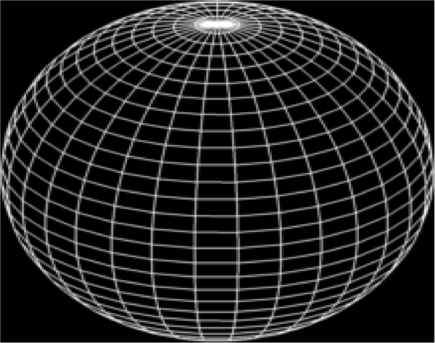

Supplement: S2 Dataset — (ZIP) [file pone.0131161.s002.zip › gt_edges_sphere.png]
